# Ten simple rules for writing scientific op-ed articles

**DOI:** 10.1371/journal.pcbi.1008187

**Published:** 2020-09-17

**Authors:** Hoe-Han Goh, Philip Bourne

**Affiliations:** 1 Institute of Systems Biology, Universiti Kebangsaan Malaysia, UKM Bangi, Selangor, Malaysia; 2 School of Data Science, University of Virginia, Charlottesville, Virginia, United States of America; Dassault Systemes BIOVIA, UNITED STATES

## Introduction

Op-eds, or opinionated editorial essays, are opinion pieces typically written for newspapers or magazines and intended for a wide audience. There are op-ed writers who specialize in writing broadly, and there are subject matter experts that focus on specific topics. Apart from other means of online outreach [1], an op-ed is an effective way to express a widely disseminated opinion about a topic. As a scientist, you get the prestige and satisfaction of expressing your point of view in a competitive, most-read section of a major publication. In the best case, it could influence decision-making and make a difference [2]. Op-eds are not like writing a scientific article nor is the process to publication the same. We offer ten simple rules as guidance, based on our experiences as op-ed writers and columnists.

## Rule 1: Be timely or timeless

The timing of an op-ed submission is an important determinant of its acceptance. Timeliness is fast-changing and can be seasonal or based on current hot topics, incidents, occasions, or events. For example, environmental issues, natural disasters, new technology launches, or breakthroughs in scientific discovery. The op-ed should be timely and predictive as to what comes next. Focus on a topic that is gaining interest and relevant to the current issues. It is an opportunity to be speculative in a way not possible with a research article.

Alternatively, be timeless. Address a subject that has yet to be adequately addressed but for which you have new opinions and ideas. Lack of diversity in science, the importance of studying both the humanities and science, technology, engineering, and mathematics (STEM) subjects, the peer review process, and research ethics are timeless examples that come to mind.

## Rule 2: Write with passion

Writing anything takes time. Why do it when it likely does not count towards your H-index or indeed promotion? The main motivation must be passion, not your scientific reputation, although well-stated opinions can help your reputation [3]. What stirs that passion? It could be a eureka moment, the desire to debunk myths, refute fake news, or a myriad of other reasons.

You are not writing a scientific paper that calls for a certain tone and relaying of the facts and conclusions in a dry and passionless way. You are stating an opinion. If you are not passionate about that opinion, don’t write it. If you are, write it in a style that reflects that passion, resonates with the reader, and has them thinking about it afterwards. That may not be easy and may take a number of iterations.

## Rule 3: Write with authority

Focus on topics in your areas of expertise that convey a strong link to your subject area. Op-ed articles should focus on one topic, so be specific. Good op-eds are based on solid research; thus, you can only write authoritatively in your own research area. You can emphasize your authority with a first-person voice based on your personal experiences so that it relates better to the readers. Op-eds are considered personal opinions of the authors; thus, editors will prefer writers with the appropriate credentials based on affiliations or knowledge of the topics.

## Rule 4: Write with persuasion

An op-ed is commonly an opinionated, one-sided argument rather than a diplomatic discussion. You need to make your arguments persuasive by supporting them with data and/or facts. Write without anger or rudeness and avoid being over-opinionated. There should be a balance between opinion and truth. Recognizable truths will improve confidence in what you have written. Previously unknown facts will add values to your writing: they may even be the reason for the op-ed in the first place. Whatever the balance, readers will be more convinced if you can relate to them using easy to understand examples or analogies.

## Rule 5: Write with insight

Your expert opinions count, so be critical and insightful in your analysis of the topic. Avoid superficial statements. Propose unusual points of view that most people have overlooked in the subject matter. Provide sound advice and propose practical actions to be taken for the general public, but do not lecture your readers. Give constructive arguments or suggestions for the stakeholders or policymakers. Conclude with a key take-home message that resonates and will be remembered (see Rule 7).

## Rule 6: Write for a general audience

The first requirement of an op-ed is that it must be readily understandable. Imagine that you are in a conversation with the general public of different ages and backgrounds. Use active voice frequently. Avoid scientific jargon if you can replace it with an everyday equivalent. Assume no a priori specialized knowledge and think from the readers’ point of view. Assuming an audience’s level of understanding helps you to identify the basic background information needed in making your arguments clear. Writing an op-ed is good training for scientists in improving their communication skills. Asking nonscientist friends to read it before submission will help you achieve readability.

## Rule 7: Write succinctly and end where you began

It should be clear what you are writing about in the first couple of sentences: the hook. You then need to end where you started, either answering the question you raised, proposing next steps, or just stating the issue(s) that remain open: the line and sinker. A good op-ed will invoke goose bumps or some other reactions at the end. Beyond the beginning and the end, it is important to keep your writing concise. Avoid obscure or flowery language, unnecessary words, and lengthy sentences. Less is more. Keep the thesis statements loud and clear for the readers. You can start the first draft by including all the details before trimming out some of the obvious statements or clichés. The quality of the final version will depend on your ability to synthesize key messages into the briefest of sentences with a logical flow. Clear writing reflects clarity of thought. Hence, think about what you want to convey and write it as simply as possible using short words. Brevity is key.

## Rule 8: Write creatively

Your piece will get extra attention from the editor if it is engaging and has an unusual point of view. The use of catchy titles, phrases, or quotes will enhance your piece. This is very different from the journal articles, which are generally matter-of-fact. Op-ed articles require creative writing techniques and narrative skills. Some figures of speech, such as metaphors and similes, can enrich language and aid understanding when applied accordingly. You can also play the devil’s advocate in arguing the rarer points or presenting your unique perspective. As a scientist, you could also be an academician, a manager, a parent, etc., with a certain origin, ethnicity, and faith. Hence, your op-ed should not be restricted to just the role of a researcher. Be flexible and creative with your different identities and various perspectives.

## Rule 9: Learn from others

Most reputable newspapers have excellent op-ed columnists. Wikipedia can help here with their list of newspaper columnists form different countries [4]. Read their work with these rules in mind. Closer to home you cannot go past the work of Sydney Brenner with his Loose Ends (later renamed False Starts) published in a scientific journal [5]. Quite simply, each one is a masterpiece that has you reading them one after another as you would a thrilling novel. As for lay publications, readers can refer to a few sources of superb op-eds [6–7] authored by some of the most followed scientists on Twitter [8].

## Rule 10: Be patient and persistent

Be aware of the lead times and be considerate of the editors’ response time. It might take a while for the editors who receive hundreds or thousands of daily submissions to respond. Or, more likely, they will not respond at all. Even if it is accepted, you might not get any reply from the paper editors. Sometimes, you only realize when it appears copyedited or published. Understand the conditions of publication, including things like liability. A shorter alternative to an op-ed is a letter to the editor with many of the same rules applied, which can also be very influential. The editors might keep the article for days or even months before publication to garner the greatest readers’ interest. It is a frustrating process, so patience is required, as is persistence. No response to multiple submissions? Keep trying and just share your output that is not published through blogs or tweets. If your opinion is a good one and valued, social media will be your friend.

## Concluding remarks

The writing of these guidelines is inspired by the recent COVID-19 pandemic, the black lives matter groundswell, and the general poor state of world politics. Never has it been a better time to write a scientific op-ed.

HHG has experience in contributing op-ed articles and as a columnist for mainstream newspapers in Malaysia.

PEB was the Founding Editor-in-Chief of this journal and, for a number of years, wrote editorials that expressed opinions relevant to this readership. He also maintains a blog, [9] where this tradition continues.
